# Left‐sided gallbladder and Chilaiditi syndrome in an infant

**DOI:** 10.1002/jpr3.12153

**Published:** 2024-12-16

**Authors:** Laura J. Bradstreet, Teresa Chapman, Jonathan O. Swanson, Katryn N. Furuya

**Affiliations:** ^1^ Divison of Pediatric Gastroenterology, Hepatology, and Nutrition American Family Children's Hospital Madison Wisconsin USA; ^2^ Department of Radiology University of Wisconsin Madison School of Medicine and Public Health Madison Wisconsin USA; ^3^ Department of Pediatrics University of Wisconsin Madison School of Medicine and Public Health Madison Wisconsin USA

**Keywords:** congenital malrotation, ectopic gallbladder, hepatic anomalies, hepatic‐diaphragmatic interposition, pediatric

## Abstract

Congenital hepatic anomalies may be associated with important intestinal abnormalities, vascular anomalies, or may be asymptomatic and discovered incidentally. Uncommon and rare anatomic liver and biliary disorders include left‐sided gallbladder, wandering liver, malrotation of the liver, and hepato‐diaphragmatic interposition of the bowel (Chilaiditi syndrome). This report describes an infant with incidentally discovered malpositioning of the gallbladder to the superior surface of the left hepatic lobe, coupled with Chilaiditi syndrome, a configuration that has not been described in the literature. Management considerations are discussed.

**Abbreviations**
ALT: alanine aminotransferase
AST: aspartate aminotransferase
CT: computerized tomography
GB: gallbladder
MRCP: magnetic resonance pancreaticocholangiography
US: ultrasound

## 1 INTRODUCTION

Congenital hepatic anomalies may be associated with intestinal abnormalities or vascular anomalies that carry surgical implications or may be asymptomatic and discovered incidentally. Here, we present a case of ectopic positioning of the gallbladder at the superior surface of the left hepatic lobe, coupled with Chilaiditi syndrome, discovered on ultrasound (US) and confirmed by computerized tomography (CT) scan in a 3‐month‐old infant who was being evaluated for abnormal vasculature in the setting of prenatally diagnosed cardiomegaly and secundum atrial septal defect.

## 2 CASE REPORT

**Figure 1:**
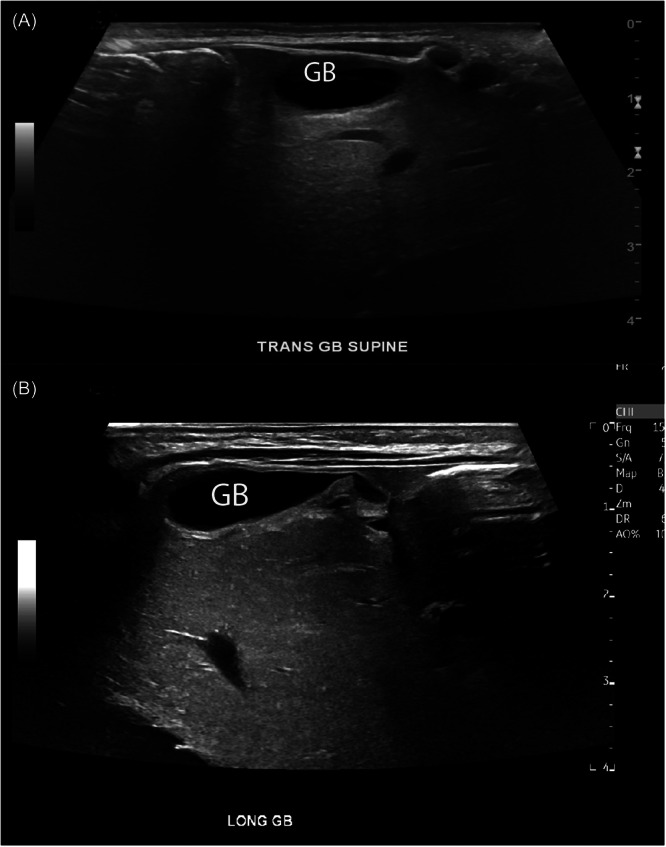
Abdominal ultrasound performed at age 1 day shows abnormal position of GB. Transverse (A) and longitudinal (B) show GB positioning at the anterior surface of the left hepatic lobe, rather than as expected under the surface of the right hepatic lobe. GB, gallbladder.

The patient is a male infant born at 39 weeks of gestation via elective repeat cesarean section and admitted to the neonatal intensive care unit for respiratory distress. Echocardiography performed for evaluation of prenatal cardiomegaly identified a large secundum atrial septal defect with left to right shunting. Additionally, prenatal sonographic identification of a direct umbilical vein connection to the right atrium and absence of the ductus venosus prompted evaluation for Abernethy malformation by abdominal US on Day 2 of life. The US did not reveal intrahepatic arteriovenous malformations, but showed an abnormal location of the gallbladder, positioned anterior to the left hepatic lobe (Figure 1A,B). No abnormality of hepatic echotexture, biliary dilatation, or liver mass was observed. Laboratory studies were notable for slightly elevated alanine aminotransferase of 47 U/L (normal 10–35) with a normal aspartate aminotransferase of 31 U/L (normal 0–83) and direct bilirubin of 0.3 mg/dL (normal 0–0.3). He was discharged home at 23 days of age.

**Figure 2:**
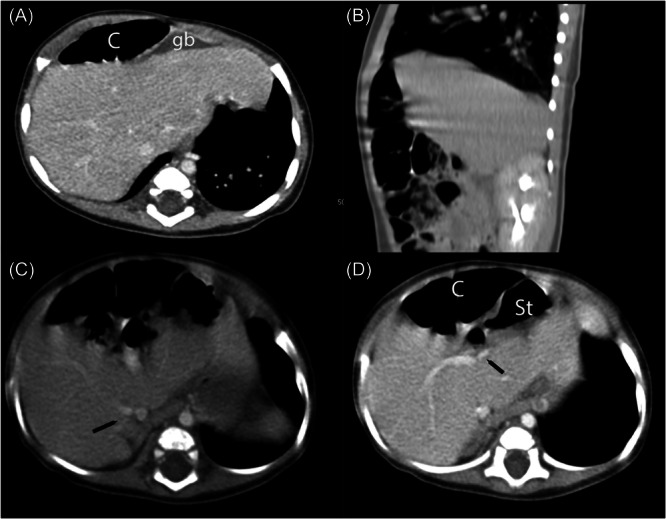
Contrast‐enhanced abdominal CT at age 4.5 months. (A) Axial image through the upper liver shows unusual position of the GB positioned to the left of midline and at the anterior surface of the liver. (B) Sagittal image demonstrates colonic interposition between the diaphragm and the liver in the right upper quadrant. (C) Axial image through the lower liver shows an accessory inferior right hepatic vein (*black arrow*), a common anatomic variant in the normal population. (D) Axial image through the mid liver shows branching of the main portal vein, with a patent but diminutive left portal vein (*black arrow*). C, colon; CT, computerized tomography; GB, gallbladder.

The patient was seen in follow‐up at 2 months of age. At that time, he appeared well, without hepatosplenomegaly. A follow‐up US done at 3.5 months of age showed the abnormal positioning of the gallbladder as initially seen, and spectral wave Doppler showed an unusual posterior‐inferior course of the proper hepatic artery, together raising concern for malrotation of the liver. A CT scan performed at age 4.5 months showed a normally sized gallbladder along the anterior surface of the left hepatic lobe (Figure 2A). CT imaging additionally showed hepato‐diaphragmatic interposition of bowel (Chilaiditi syndrome), an accessory right inferior hepatic vein, diminutive but patent left portal vein, and otherwise normal vascular anatomy (Figure 2B–D). Spleen and kidneys were normal. The patient has remained asymptomatic and is currently meeting his growth parameters and developmental milestones at 8 months of age. We have advised the parents that Chilaiditi syndrome is a benign condition; however, on plain abdominal X‐rays, it may appear as free air, which may then result in unwarranted surgeries. Similarly, liver biopsies performed in patients with Chilaiditi syndrome may result in perforation of the malpositioned bowel. This diagnosis also carries implications for patients undergoing cholecystectomy in terms of the surgical approach.

## 3 DISCUSSION

Congenital anomalous positioning of the gallbladder is uncommon and may be described by various terms, including transposition of the gallbladder, left‐sided gallbladder, or ectopic gallbladder.^1^, ^2^ A left‐sided gallbladder has a reported prevalence of 0.1%–0.7%^1^, ^2^ and refers to a gallbladder positioned left of the round ligament with an aberrant attachment to the undersurface of segment 3. Describing this anomaly, the middle hepatic vein courses to the right of the gallbladder and the round ligament originates from the left portal vein.^2^ This matches the anatomy of our infant here, except that the gallbladder has an anomalous position on the superior surface of the left hepatic lobe, rather than the undersurface. The observed small size of the left portal vein was subjective and is of uncertain significance given its patency. This biliary tree anomaly presumably arises early, in the fourth week of embryologic development, as the hepatic diverticulum extends from the ventral foregut.^3^ Left‐sided gallbladder becomes evident typically with symptomatic cholelithiasis, prompting US imaging, and it carries implications for the surgical approach to cholecystectomy, but the anomalous positioning in and of itself does not cause abdominal symptoms and does not merit a surgical intervention.

Chilaiditi syndrome (also referred to by some as Chilaiditi sign in asymptomatic cases), refers to hepatic‐diaphragmatic interposition of the bowel and is an uncommon anatomic finding (incidence of 0.025%–0.28% worldwide), wherein the transverse colon, or less commonly small bowel, lies between the right hemidiaphragm and the right hepatic lobe.^4^ The observation of colon positioned between the diaphragm and liver radiographically was first described in 1910 by a radiologist named Demetrius Chilaiditi.^5^ Predisposing factors for Chilaiditi syndrome may include a combination of anatomic anomalies, including a redundant colon with a long mesentery, relaxation or elongation of the hepatic suspensory ligaments, or segmental agenesis of the liver.^6^, ^7^ Although most patients remain asymptomatic, abdominal or chest pain has been attributed to this anatomy. An association between Chilaiditi syndrome and colonic volvulus has been described mostly in adults^8^, ^9^ as well as in a child.^10^ This anatomical variant also carries the risk of confusion with pneumoperitoneum or diaphragmatic hernia on radiographs. Conservative management is often sufficient in a child with symptomatic Chilaiditi syndrome.^11^

The unusual appearance of the liver anatomy initially concerned hepatic malrotation, with the ventral surface of the left hepatic lobe perhaps being rotated upward in the abdomen. In adults, few cases have been described as incidental discoveries either at postmortem assessment or as irrelevant to their symptoms that prompted diagnostic imaging.^12^, ^13^ Features on prior descriptions include the following: normal situs, rightward and inferior rotation of the liver, rendering the gallbladder facing posteriorly, with normal relation of vascular and biliary systems to liver segments, and a normal spleen.^12^ Our patient's liver and gallbladder anatomy did not match these descriptions for hepatic malrotation. Another consideration was a wandering or hypermobile liver, also referred to as hepatoptosis, hepatic dislocation, and hepatic ectopia, which has been described in both adults and children.^14^, ^15^ This entity may develop when the hepatic suspensory ligaments fail to develop normally, allowing the liver to “wander” out of the normal position. The majority of published cases of wandering liver describe asymptomatic cases discovered in adulthood, as well as patients with chronic or recurrent abdominal discomfort, suggestive of colonic obstruction. Rarely, wandering liver presents as concomitant midgut volvulus prompting emergent surgery. Symptomatic patients with this entity require surgical hepatopexy.^15^ In our patient's case, however, the liver has remained in the right hemiabdomen.

Anatomical variations of the liver can create diagnostic challenges and raise questions about appropriate clinical management. To the best of our knowledge, this case of left‐sided gallbladder positioned superior to the left hepatic lobe coupled with hepato‐diaphragmatic interposition of the bowel has not yet been described in the literature. The developmental etiology may overlap with prior descriptions of hepatic malrotation, likely extremely rare, and can be expected to remain asymptomatic. For this infant, current management regards congenital heart disease only, and should any gastroenterology symptoms arise, further evaluation with repeat US and magnetic resonance pancreaticocholangiography will be pursued.

## CONFLICT OF INTEREST STATEMENT

The authors declare no conflicts of interest.

## ETHICS STATEMENT

Written informed consent was obtained from the guardian of the patient, as the patient is underage, for the publication of their details in this case study.
